# Beyond Precision: A Multidimensional Framework for Selecting Genetic Medicine Platforms

**DOI:** 10.3390/cells15181647

**Published:** 2026-09-11

**Authors:** Jared Wieland, Peyton Jackson, William Penrod, Spencer Nadauld, Jared Barrott

**Affiliations:** 1 Department of Cell Biology & Physiology, Brigham Young University, Provo, UT 84602, USA; jaw14@byu.edu (J.W.); willp34@student.byu.edu (W.P.); snadauld@student.byu.edu (S.N.); 2 Indel Bioinnovations, Provo, UT 84602, USA; peyton.jackson@indelbioinnovations.com; 3 Simmons Center for Cancer Research, Brigham Young University, Provo, UT 84602, USA; 4 School of Medicine, Brigham Young University, Provo, UT 84602, USA

**Keywords:** gene therapy, genetic medicine, genome editing, CRISPR-Cas, base editing, prime editing, gene delivery, genomic safety

## Abstract

Gene therapy is undergoing continued clinical translation and technological development. This progress has been marked by regulatory approvals and broadened therapeutic indications across genetic, metabolic, and oncologic diseases and disorders. The field has evolved over decades from early viral-mediated gene addition to approaches capable of targeted editing, regulation, or replacement of genetic information. These systems include base and prime editors, epigenetic modulators, CRISPR-Cas, RNA therapeutics and programmable integration platforms. When paired with increasingly sophisticated viral and nonviral delivery strategies, these technologies enable greater control over tissue targeting, duration of activity, and therapeutic exposure. Recent clinical successes, including approved ex vivo CRISPR-based therapies for hemoglobinopathies, in vivo CRISPR editing for transthyretin amyloidosis, and emerging clinical applications of base and prime editing, provide growing clinical evidence for the feasibility of genetic medicines. However, technological advancement has also made platform selection increasingly complex. Therapeutic performance is determined not by editing efficiency alone, but by the interaction among genetic precision, temporal control, dosage tunability, delivery efficiency, durability, and disease-specific safety requirements. A molecularly efficient platform may still have limited therapeutic value if it cannot reach the disease-relevant cell population at sufficient and safe exposure. In this review, we examine recent technological and clinical advances in genetic medicine with particular emphasis on developments during the past approximately five years. We propose a multidimensional framework in which gene therapy platforms are evaluated according to three intrinsic properties—genetic precision, temporal control, and dosage tunability—while delivery, clinical maturity, and disease context act as major translational constraints. This framework highlights that no single platform is universally optimal; rather, successful therapeutic design depends on matching the biological characteristics of the intervention to the requirements of the disease and target tissue. Remaining challenges in extrahepatic delivery, genomic safety, immunogenicity, manufacturing, and long-term monitoring remain important determinants of broader clinical implementation.

## 1. Introduction

Gene therapy refers broadly to therapeutic delivery, gene modification, or transient regulation of genetic material to prevent or treat diseases [1,2,3,4,5,6,7,8,9,10]. This definition encompasses a wide range of approaches including the addition or silencing of functional genes, correction of pathogenic mutations, and regulation of gene expression at the DNA or RNA level [5,6,7,8,9,10,11,12,13,14,15,16,17,18,19,20,21,22,23,24,25,26,27,28,29]. Gene therapies can address disease-associated genetic mechanisms and, in selected indications, provide durable therapeutic benefit [1,2,3,4,11,12,13,14].

The evolution of gene therapy began with the modification of viruses [11,12,13,14,30,31,32,33,34,35,36,37,38,39]. Viral gene addition strategies (primarily utilizing retroviral and lentiviral techniques) as well as Adeno-associated viral vectors (AAVs) became the basis for introducing genetic material [11,12,13,14,30,31,32,33,34,35,36,37,38,39]. While these approaches enabled early clinical successes, they were constrained by limitations in safety, control, and precision [11,12,13,14,40,41,42,43,44,45,46,47,48]. Insertional mutagenesis, immune responses, and variable post-treatment gene expression illustrate the need for more refined technologies [40,41,42,43,44,45,46,47,48].

In the last decade, particularly the last five years, the field has placed increasing emphasis on targeted genetic modification and control of therapeutic activity [10,15,16,17,18,19,20,21,22,23,24,25,26,27,28,29,49,50,51,52,53,54,55,56,57,58,59,60,61,62,63,64,65,66,67,68,69,70,71]. The advent of tools for genomic editing such as zinc finger nucleases (ZFNs), transcription activator-like effector nucleases (TALENs), and CRISPR-Cas systems has enabled targeted modification of genetic sequences [10,15,16,17,18,19,20,21,22]. Most recent innovations include base editing, prime editing, and control of genetic function while avoiding inefficient double-stranded DNA breaks (DSBs) repaired by homologous recombination (HR) [23,24,25,26,27,28,29,49,50,51,52,53,54,55,56,57,58,59,60,61,62,63,64,65,66,67,68,69,70,71].

This narrative review emphasizes primary clinical and translational studies published through August 2026, with selected foundational studies included to establish technological development. Literature was identified through searches of PubMed and major peer-reviewed journals using terms related to gene therapy, genome editing, base editing, prime editing, epigenome editing, viral and nonviral delivery, and clinical translation. Literature searches were last updated on 31 August 2026; priority was given to primary clinical and translational studies, with foundational studies retained when needed to define platform mechanisms. Regulatory guidance from the U.S. Food and Drug Administration was included where directly relevant to genome-editing development. This review focuses on the recent technological and clinical advances that have reshaped the landscape of gene therapy [1,2,3,4,5,6,7,8,9,10,15,16,17,18,19,20,21,22,23,24,25,26,27,28,29,30,31,32,33,34,35,36,37,38,39,40,41,42,43,44,45,46,47,48,49,50,51,52,53,54,55,56,57,58,59,60,61,62,63,64,65,66,67,68,69,70,71,72,73,74,75,76,77,78,79,80,81,82,83,84,85,86,87,88]. We emphasize developments in genome editing technologies, delivery strategies, and clinical translation, with particular attention to progress made over the past approximately five years [1,2,3,4,23,24,25,26,27,28,29,30,31,32,33,34,35,36,37,38,39,40,41,42,43,44,45,46,47,48,49,50,51,52,53,54,55,56,57,58,59,60,61,62,63,64,65,66,67,68,69,70,71,72,73,74,75,76,77,78,79,80,81,82,83,84,85,86,87,88]. Rather than considering these technologies only as a chronological series of increasingly advanced platforms, we evaluate them within a common therapeutic design framework. Specifically, we consider genetic precision, temporal control, and dosage tunability as three intrinsic properties of a genetic intervention, while delivery efficiency, tissue accessibility, disease biology, and clinical maturity determine whether those properties can be translated into therapeutic benefit. This framework provides a basis for comparing technologies that otherwise differ substantially in mechanism and highlights an increasingly important principle of genetic medicine: the most technically precise platform is not necessarily the most clinically appropriate platform for every disease. This review does not attempt comprehensive coverage of all nucleic acid–based therapeutics. RNA therapeutics are included selectively because their transient and repeat-dose characteristics provide an informative comparator for evaluating temporal control and dosage tunability across genetic medicine platforms.

### 1.1. Defining the Therapeutic Design Space

The increasing diversity of genetic technologies creates a challenge that extends beyond determining whether a particular gene can be modified. Modern therapeutic development requires selection among platforms that differ in permanence, precision, reversibility, delivery requirements, immune consequences, and clinical maturity [5,6,7,8,9,10,15,16,17,18,19,20,21,22,23,24,25,26,27,28,29,30,31,32,33,34,35,36,37,38,72,73,74,75,76,77,78,79,80,81,82]. A useful comparison therefore requires consideration not only of what genetic change a technology can produce, but also how, where, for how long, and to what extent that change is produced.

To organize these differences, this review considers three intrinsic dimensions of therapeutic control: genetic precision, temporal control, and dosage tunability. Genetic precision describes the ability to produce the intended molecular and functional outcome while limiting unintended genomic or biological alterations [18,19,20,21,22,40,41,42,43]. Temporal control describes the duration and timing of therapeutic activity, including both the persistence of the therapeutic machinery and the durability of the resulting biological effect [23,24,25,26,27,28,29,74,75,76,77]. Dosage tunability describes the ability to regulate the magnitude of gene expression or therapeutic activity before or after treatment [5,6,7,8,9,23,28,29].

These dimensions should not be considered independently of delivery. Delivery determines whether a therapeutic platform reaches the appropriate organ, cell population, and intracellular compartment at sufficient concentration to produce the desired effect [30,31,32,33,34,35,36,37,38,72,73,74,75,76,77,78,79,80,81,82]. Likewise, clinical maturity and disease context influence the acceptable balance among permanence, efficiency, safety, and reversibility. A permanent intervention may be advantageous for a severe monogenic disease in which lifelong correction is desirable, but less appropriate for a common disease in which even a small safety risk could affect a much larger treatment population. Conversely, a transient RNA-based intervention may require repeated administration but provide greater control over exposure and reversibility [5,6,7,8,9].

This conceptual framework does not assume that technological progress follows a simple progression from less advanced to more advanced platforms. Rather, gene addition, genome editing, transcriptional regulation, RNA-based therapeutics, and programmable integration occupy different regions of a broader therapeutic design space. Their clinical value depends on how well their mechanistic properties align with the biological requirements, delivery constraints, and acceptable risk profile of a particular indication. These relationships are examined in greater detail later in the review through a multidimensional comparison of genetic medicine platforms.

### 1.2. From Viral Gene Addition to Precision Medicine

Gene therapies were originally developed around the concept of gene addition, in which a functional copy of a defective or missing gene is delivered into patient cells to restore biological function [11,12,13,14]. This strategy proved particularly attractive for monogenic disorders, where restoration of a single gene could produce clinically meaningful benefit in selected monogenic disorders [11,12,13,14].

Clinical translation of these early approaches was enabled primarily through viral vectors, particularly adeno-associated virus (AAV) and lentiviral platforms [11,12,13,14,30,31,32,33,34,35,36,37,38,39]. AAV vectors became especially valuable for in vivo delivery because of their ability to transduce post-mitotic tissues and support long-term episomal expression [30,33,36,37,38,39]. Lentiviral vectors, in contrast, became central to ex vivo gene therapy because of their ability to establish stable genomic integration in dividing cells [11,12,13,14]. Together, these platforms facilitated durable therapeutic outcomes in retinal disease, spinal muscular atrophy, inherited immunodeficiencies, and hemoglobinopathies [11,12,13,14].

Early FDA-approved examples demonstrated the clinical feasibility of durable therapeutic gene expression or genetic modification in humans [11,12,13,14]. These successes established proof of concept that genetic diseases could be treated through direct modification of cellular genetic content [11,12,13,14].

Despite these advances, viral gene addition strategies revealed several important limitations that continue to shape modern gene therapy development [40,41,42,43,44,45,46,47,48]. Viral vectors can trigger both innate and adaptive immune responses, particularly in the setting of pre-existing anti-AAV immunity or high systemic vector doses [44,45,46,47,48]. In addition, conventional gene addition provides limited control over expression level, timing, and cellular specificity, all of which can influence therapeutic efficacy and safety [30,38,39]. Therapeutic benefit often depends on maintaining gene expression within a narrow physiological range, as insufficient expression may fail to correct disease while excessive expression may produce toxicity or unintended biological effects [30,38,39].

Integrating vectors such as retroviral and lentiviral systems also raised concerns regarding insertional mutagenesis and clonal expansion [40,41,42]. These risks are particularly relevant when integration occurs near proto-oncogenes or critical regulatory elements capable of influencing cellular proliferation [40,41]. Although modern self-inactivating lentiviral systems have substantially improved safety profiles, insertion-site monitoring remains an important component of clinical development and long-term follow-up [12,13,14].

**Figure 1:**
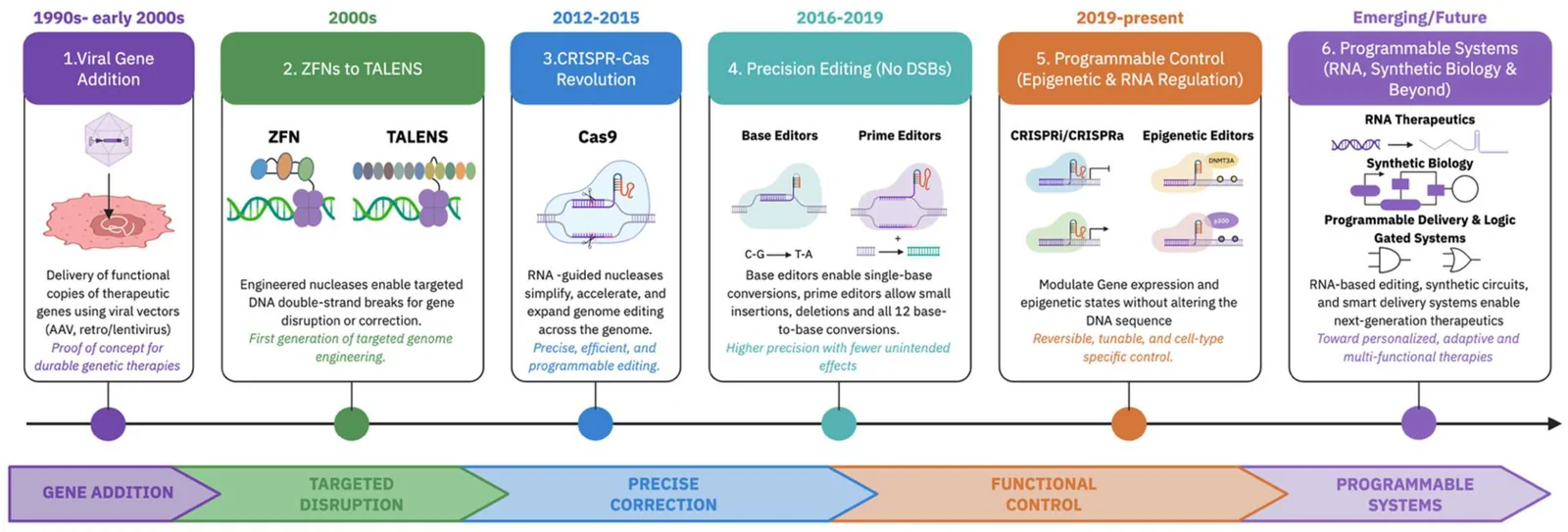
Condensed timeline of genetic medicine development. Schematic overview of major technological transitions in genetic medicine, progressing from early viral-mediated gene addition to programmable genome engineering and gene regulation. Early approaches relied predominantly on viral vectors for delivery of functional gene copies, followed by the development of targeted nucleases such as zinc finger nucleases (ZFNs) and transcription activator-like effector nucleases (TALENs). The introduction of CRISPR-Cas systems enabled RNA-guided genome editing and accelerated the development of more precise platforms, including base and prime editing, which can modify genetic information with reduced reliance on double-strand DNA breaks. More recent approaches incorporate programmable transcriptional and epigenetic regulation, RNA-based therapeutics, synthetic biology, and targeted integration systems. The timeline is intended as a conceptual representation of major developments rather than an exhaustive chronology; individual technologies overlap in their periods of development and clinical translation. Abbreviations: CRISPR, clustered regularly interspaced short palindromic repeats; DSB, double-strand break; RNA, ribonucleic acid; TALEN, transcription activator-like effector nuclease; ZFN, zinc finger nuclease.

The limitations of viral gene addition motivated a shift from gene supplementation toward direct genome modification and regulation [10,15,16,17,18,19,20,21,22]. Rather than asking whether a missing gene could be supplied, researchers increasingly focused on whether disease-causing genetic defects could be repaired, disrupted, or transcriptionally controlled at their endogenous loci [10,15,16,17,18,19,20,21,22]. This transition drove the development of programmable genome engineering technologies capable of targeting specific genomic sequences [10,15,16,17,18,19,20,21,22]. This historical progression is summarized schematically (Figure 1).

Among the earliest programmable editing systems were zinc finger nucleases (ZFNs), which combined engineered zinc-finger DNA-binding domains with the FokI nuclease to generate targeted double-strand DNA breaks [10,15]. Transcription activator-like effector nucleases (TALENs) subsequently improved targeting flexibility by utilizing modular DNA-binding domains that were easier to design and customize [15,16]. Both platforms demonstrated that genomic DNA could be modified at predetermined loci rather than through random integration events [10,15,16]. However, the complexity of protein engineering limited their scalability and widespread adoption [10,15].

The emergence of CRISPR-Cas systems accelerated the development and adoption of programmable genome editing [10,15,16,17,18,19,20,21,22]. In CRISPR-Cas9 editing, a guide RNA directs the nuclease to a complementary genomic sequence, enabling programmable cleavage through a comparatively simple and adaptable framework [10,15,16,22]. Because target specificity is determined primarily by guide RNA sequence rather than engineered protein domains, new genomic targets can be rapidly identified and tested [10,15,16]. This simplicity accelerated both basic research and therapeutic development across a broad range of disease areas [1,2,3,4,10,15,16,17,18,19,20,21,22].

Clinically, CRISPR shifted gene therapy beyond transgene delivery toward gene correction, gene disruption, regulatory editing, and cell-state engineering [1,2,3,4,10,15,16,17,18,19,20,21,22]. The approval of exagamglogene autotemcel (Casgevy) for sickle cell disease and β-thalassemia represented the first regulatory approval of a CRISPR-edited therapy for these indications and provided clinical validation of ex vivo genome editing [2,3]. These achievements demonstrated that targeted modification of endogenous genomic loci could produce durable therapeutic benefit in human patients [1,2,3].

This transition expanded therapeutic strategies beyond conventional transgene delivery [10,15,16,17,18,19,20,21,22]. In classical viral gene addition, the therapeutic payload is typically external to the endogenous disease locus [11,12,13,14]. In genome editing, therapeutic intervention occurs directly within disease-relevant genes or regulatory elements that control disease biology [1,2,3,4,10,15,16,17,18,19,20,21,22]. Such interventions may correct pathogenic mutations, disrupt harmful genes, reactivate fetal hemoglobin expression, or modify immune-cell function to improve therapeutic outcomes [1,2,3,4,89,90,91,92,93].

Recent technological advances have extended genome engineering beyond conventional double-strand-break-mediated editing [23,24,25,26,27,28,29,49,50,51,52,53,54,55,56,57,58,59,60,61,62,63,64,65,66,67,68,69,70,71]. Base editors enable precise single-nucleotide conversion without generating canonical double-strand DNA breaks, reducing the risk of large-scale genomic alterations [49,50,51,52,53,54,55,56,57,58,59]. Prime editors further expand editing capabilities by enabling targeted insertions, deletions, and sequence rewriting through a reverse-transcriptase-mediated mechanism [56,60,61,62,63,64,65,66,67,68,69,70,71]. Additional platforms, including CRISPR interference (CRISPRi), CRISPR activation (CRISPRa), and epigenome editing systems, permit programmable regulation of gene expression without altering underlying DNA sequence [23,24,25,26,27,28,29].

Together, these innovations have moved gene therapy beyond simple gene replacement toward programmable medicine, in which therapeutic design can be tailored to a patient’s specific mutation, target tissue, desired duration of activity, and acceptable safety profile [23,24,25,26,27,28,29,49,50,51,52,53,54,55,56,57,58,59,60,61,62,63,64,65,66,67,68,69,70,71,74]. The transition from viral gene addition to precision genome engineering therefore represents not merely technological advancement but a shift toward therapeutic strategies that can be selected according to disease mechanism, target tissue, desired durability, and acceptable risk [1,2,3,4,10,15,16,17,18,19,20,21,22].

### 1.3. Current Genome Engineering Technologies

**Figure 2:**
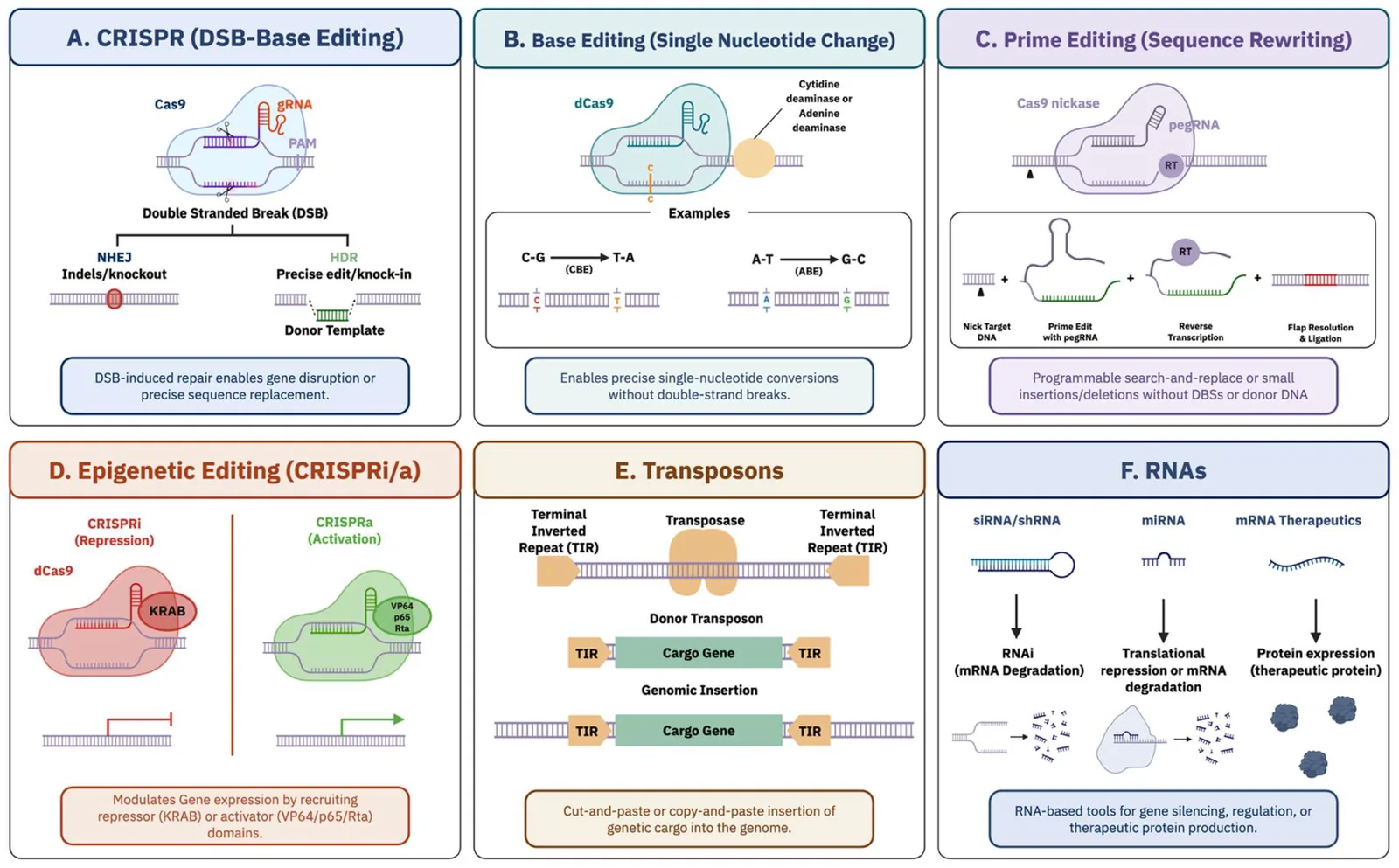
Current genome engineering and genetic medicine technologies. Schematic comparison of major contemporary platforms used to modify, regulate, or deliver genetic information. CRISPR-Cas nuclease editing introduces targeted double-strand DNA breaks that are repaired through endogenous pathways such as non-homologous end joining (NHEJ) or homology-directed repair (HDR), enabling gene disruption or sequence modification. Base editors catalyze defined nucleotide conversions without generating canonical double-strand breaks, whereas prime editors use a Cas nickase–reverse transcriptase complex and prime-editing guide RNA (pegRNA) to support programmable sequence rewriting, including selected substitutions, insertions, and deletions. Epigenetic and transcriptional editing systems, including CRISPR interference (CRISPRi) and CRISPR activation (CRISPRa), modulate gene expression without altering the underlying DNA sequence. Transposon-based systems enable genomic integration of larger genetic cargos, while RNA-based therapeutics act at the level of transcript abundance, translation, or transient protein expression. The figure is intended as a mechanistic overview rather than a comprehensive representation of all available platforms or their relative clinical maturity. Abbreviations: Cas, CRISPR-associated protein; CRISPR, clustered regularly interspaced short palindromic repeats; CRISPRa, CRISPR activation; CRISPRi, CRISPR interference; DSB, double-strand break; HDR, homology-directed repair; mRNA, messenger RNA; NHEJ, non-homologous end joining; pegRNA, prime-editing guide RNA; RT, reverse transcriptase; shRNA, short hairpin RNA; siRNA, small interfering RNA; miRNA, microRNA.

Advances in genome engineering have transformed gene therapy from a field primarily focused on gene addition into one capable of modifying, rewriting, and regulating genetic information with greater control over target sequence and therapeutic mechanism [10,15,16,17,18,19,20,21,22,23,24,25,26,27,28,29,49,50,51,52,53,54,55,56,57,58,59,60,61,62,63,64,65,66,67,68,69,70,71]. At the same time, genetic medicine has expanded beyond permanent DNA sequence modification to include transient and reversible regulation of gene expression [5,6,7,8,9,10,15,16,17,18,19,20,21,22,23,24,25,26,27,28,29]. For conceptual clarity, these approaches can be divided into related but mechanistically distinct categories: conventional gene transfer, genome editing, transcriptional or epigenetic regulation, RNA-based therapeutics, and programmable genomic integration. Each platform possesses distinct mechanistic advantages, limitations, delivery requirements, and levels of clinical maturity that influence its suitability for different therapeutic contexts [23,24,25,26,27,28,29,49,50,51,52,53,54,55,56,57,58,59,60,61,62,63,64,65,66,67,68,69,70,71,72,73,74,75,76,77,78,79,80,81]. The major contemporary platforms and their mechanisms are compared schematically (Figure 2).

CRISPR-Cas systems remain among the most widely used platforms in contemporary genome engineering [10,15,16,17,18,19,20,21,22]. These RNA-guided nucleases, most commonly Cas9 and Cas12, introduce site-specific double-strand DNA breaks that are subsequently repaired through endogenous cellular pathways such as non-homologous end joining (NHEJ) and homology-directed repair (HDR) [10,15,16,22]. NHEJ frequently results in insertions or deletions that disrupt gene function, whereas HDR can facilitate precise sequence correction or insertion when an appropriate donor template is available [10,15,16]. Compared with earlier programmable nucleases, this relative simplicity contributed to the broad adoption of CRISPR applications in both research and clinical settings [10,15,16,17,18,19,20,21,22].

Clinical implementation of CRISPR editing has been achieved through both ex vivo and in vivo therapeutic approaches [1,2,3,4,10,15,16,17,18,19,20,21,22]. Ex vivo strategies, such as modification of hematopoietic stem cells for treatment of sickle cell disease and β-thalassemia, permit extensive quality-control assessment before reinfusion into patients [2,3,4]. In contrast, in vivo editing has shown particular promise in the liver, where efficient delivery technologies facilitate direct targeting of hepatocytes [1,49,50,51,74]. One notable example is CRISPR-mediated disruption of the transthyretin (TTR) gene, which produced substantial reductions in circulating TTR protein levels in patients with transthyretin amyloidosis [1]. Genome-editing strategies targeting PCSK9 initially demonstrated durable reductions in circulating cholesterol in preclinical models [49,50,51], and have subsequently progressed to clinical evaluation with in vivo base editing [94].

The clinical utility of CRISPR systems depends heavily on effective delivery strategies [72,73,74,75,76,77,78,79,80,81]. CRISPR components may be delivered as plasmid DNA, messenger RNA (mRNA), purified protein, or ribonucleoprotein (RNP) complexes [74,75,76,77]. The chosen delivery format influences both the duration of nuclease activity and overall safety profile [74,75,76,77]. RNP delivery enables transient exposure and may reduce off-target editing, whereas mRNA-based approaches offer scalability and compatibility with nonviral delivery platforms such as lipid nanoparticles [74,75,76,77,78,79,80]. Despite these advantages, double-strand-break-dependent editing remains associated with several risks, including activation of DNA damage responses, unintended insertions or deletions, chromosomal rearrangements, and large genomic deletions [40,41,42,43]. Dependence on endogenous DNA repair pathways may further limit editing precision, particularly in non-dividing cells where HDR activity is inefficient [10,15,16,40,41,42].

To address several limitations of double-strand-break-dependent editing, second-generation genome editing technologies have been developed that minimize or eliminate the need for double-strand DNA breaks [49,50,51,52,53,54,55,56,57,58,59,60,61,62,63,64,65,66,67,68,69,70,71]. Base editing enables direct conversion of one nucleotide into another through fusion of catalytically modified CRISPR proteins with nucleotide deaminases [49,50,51,52,53,54,55,56,57,58,59]. This approach permits precise correction of point mutations without generating conventional double-strand breaks and can therefore reduce dependence on error-prone double-strand-break repair pathways [49,50,51,52,53,54,55,56,57,58,59]. Base editing has demonstrated therapeutic potential in preclinical models of hypercholesterolemia, Hutchinson-Gilford progeria syndrome, sickle cell disease, and other genetic disorders [50,51,52,53].

Importantly, avoidance of a conventional double-strand break does not eliminate the need for genomic safety assessment. Base editors possess characteristic error profiles that can include bystander editing within the activity window, guide-dependent or guide-independent off-target activity, and unwanted editing outcomes that vary according to editor architecture, target sequence, and cellular context [49,50,51,52,53,54,55,56,57,58,59]. Precision should therefore be evaluated according to the complete distribution of intended and unintended products rather than simply the absence of double-strand DNA cleavage.

Prime editing further expands genome engineering capabilities by enabling targeted sequence rewriting, including insertions, deletions, and all possible base substitutions [56,60,61,62,63,64,65,66,67,68,69,70,71]. Prime editors utilize a Cas nickase fused to a reverse transcriptase and are guided by specialized prime-editing guide RNAs (pegRNAs) that encode the desired genetic change [56,60,61,62]. Prime editing enables a broader range of programmed sequence alterations than conventional base editing, avoiding many of the limitations associated with double-strand-break-mediated repair [60,61,62,63,64,65,66,67,68,69,70,71].

Recent advances have improved prime editing efficiency, expanded editing scope, and demonstrated therapeutic efficacy in animal models of metabolic, hematologic, and ocular disease [64,65,66,67,68,69,70,71]. Prime editing has also entered early clinical application, although human evidence remains limited. PM359, an autologous CD34+ hematopoietic stem-cell therapy using prime editing to correct the common NCF1 deletion associated with p47phox-deficient chronic granulomatous disease, has produced initial evidence of engraftment and restored NADPH oxidase activity in treated participants [95]. These early findings provide important clinical proof of principle while also emphasizing the need for longer follow-up and larger patient cohorts before conclusions regarding durability and long-term safety can be established.

As with all genome-editing systems, prime editing introduces platform-specific considerations. Editing efficiency varies according to target sequence and cell type, and unintended products may arise through pegRNA-dependent events, nick-induced repair, or interactions between the reverse transcriptase and endogenous DNA repair processes [56,60,61,62,63,64,65,66,67,68,69,70,71]. Thus, the principal advantage of base and prime editing is not the elimination of genomic risk but the ability to produce different classes of genetic alteration with a distinct and potentially more favorable spectrum of editing outcomes.

Beyond direct sequence modification, epigenetic and transcriptional editing technologies permit regulation of gene expression without altering DNA sequence [23,24,25,26,27,28,29]. These approaches typically employ catalytically inactive CRISPR proteins fused to transcriptional activators, repressors, or epigenetic modifiers [23,24,25,26,27,28,29]. CRISPR activation (CRISPRa) systems increase gene expression, whereas CRISPR interference (CRISPRi) systems suppress transcription through targeted recruitment of regulatory proteins [23,24,25,26,27,28,29]. By targeting promoters, enhancers, or other regulatory elements, these technologies provide programmable control over gene activity while preserving genomic sequence integrity [23,24,25,26,27,28,29].

A potential advantage of epigenetic editing is reversible or tunable regulation of gene expression [23,24,25,26,27,28,29]. Such approaches may be particularly valuable for diseases in which transient modulation of gene dosage is preferable to permanent genomic alteration [23,24,25,26,27,28,29]. Emerging studies have also demonstrated the feasibility of delivering CRISPR-based epigenome editors through transient RNA or ribonucleoprotein platforms, further enhancing their therapeutic flexibility [28,29].

RNA-based therapeutics are considered here as a complementary class of genetic medicine because their transient effects and potential for repeat dosing provide an informative contrast to permanent DNA-modifying approaches [5,6,7,8,9]. Small interfering RNAs (siRNAs) and short hairpin RNAs (shRNAs) enable targeted degradation of messenger RNA transcripts, thereby reducing expression of disease-associated genes [5,6,7,8,9]. Conversely, mRNA therapeutics allow transient production of therapeutic proteins without permanent genomic modification [72,73]. These approaches have already achieved clinical success in diseases such as hereditary transthyretin amyloidosis, familial hypercholesterolemia, primary hyperoxaluria, and acute intermittent porphyria [5,6,7,8,9]. Because their molecular effects are transient, RNA-based therapies avoid permanent genomic modification and can permit repeat dosing, although safety remains product- and delivery-dependent [5,6,7,8,9].

Another rapidly developing class of genome engineering technologies involves transposon-based and programmable integration-based systems [83,84,85,86,87,88]. Platforms such as Sleeping Beauty and PiggyBac facilitate stable integration of genetic cargo into the genome through transposase-mediated insertion mechanisms [83,84,85,86,87]. These systems can accommodate relatively large genetic cargos compared with vectors such as AAV [83,84,85]. Because the transposase itself can be supplied transiently while the integrated genetic cargo remains stable, these systems separate the duration of enzymatic exposure from the durability of the resulting genomic modification.

This distinction is important when considering temporal control. Degradation of the transposase limits the period during which new integration events can occur, but it does not reverse integrations that have already been established. Furthermore, conventional transposon systems do not provide complete site-specific control, and multiple or unintended integrations can create insertional and genomic safety concerns that require careful characterization [83,84,85,86,87]. Repeat administration is therefore technically possible in some nonviral configurations but should not be interpreted as equivalent to the titratable repeat dosing characteristic of transient RNA therapeutics.

More recently, CRISPR-associated transposase (CAST) systems have emerged that combine RNA-guided targeting with programmable DNA integration [83,84,85,86,87,88]. These technologies aim to increase positional control while retaining the ability to insert relatively large genetic cargos. Although recent engineering has enabled increasingly efficient targeted integration, most such platforms remain substantially earlier in translational development than established viral gene-transfer or nuclease-based genome-editing approaches [83,84,85,86,87,88]. Their future therapeutic value will depend on demonstrating predictable insertion profiles, genomic safety, efficient delivery, and durable function in clinically relevant human tissues.

Collectively, modern genome engineering technologies illustrate the diversification of therapeutic strategies available for genetic medicine [10,15,16,17,18,19,20,21,22,23,24,25,26,27,28,29,49,50,51,52,53,54,55,56,57,58,59,60,61,62,63,64,65,66,67,68,69,70,71,83,84,85,86,87,88]. The field has progressed from reliance on permanent viral gene addition toward a spectrum of programmable approaches capable of altering, regulating, or replacing genetic information according to disease-specific requirements [10,15,16,17,18,19,20,21,22,23,24,25,26,27,28,29,49,50,51,52,53,54,55,56,57,58,59,60,61,62,63,64,65,66,67,68,69,70,71].

### 1.4. Summary of Delivery Technologies

**Figure 3:**
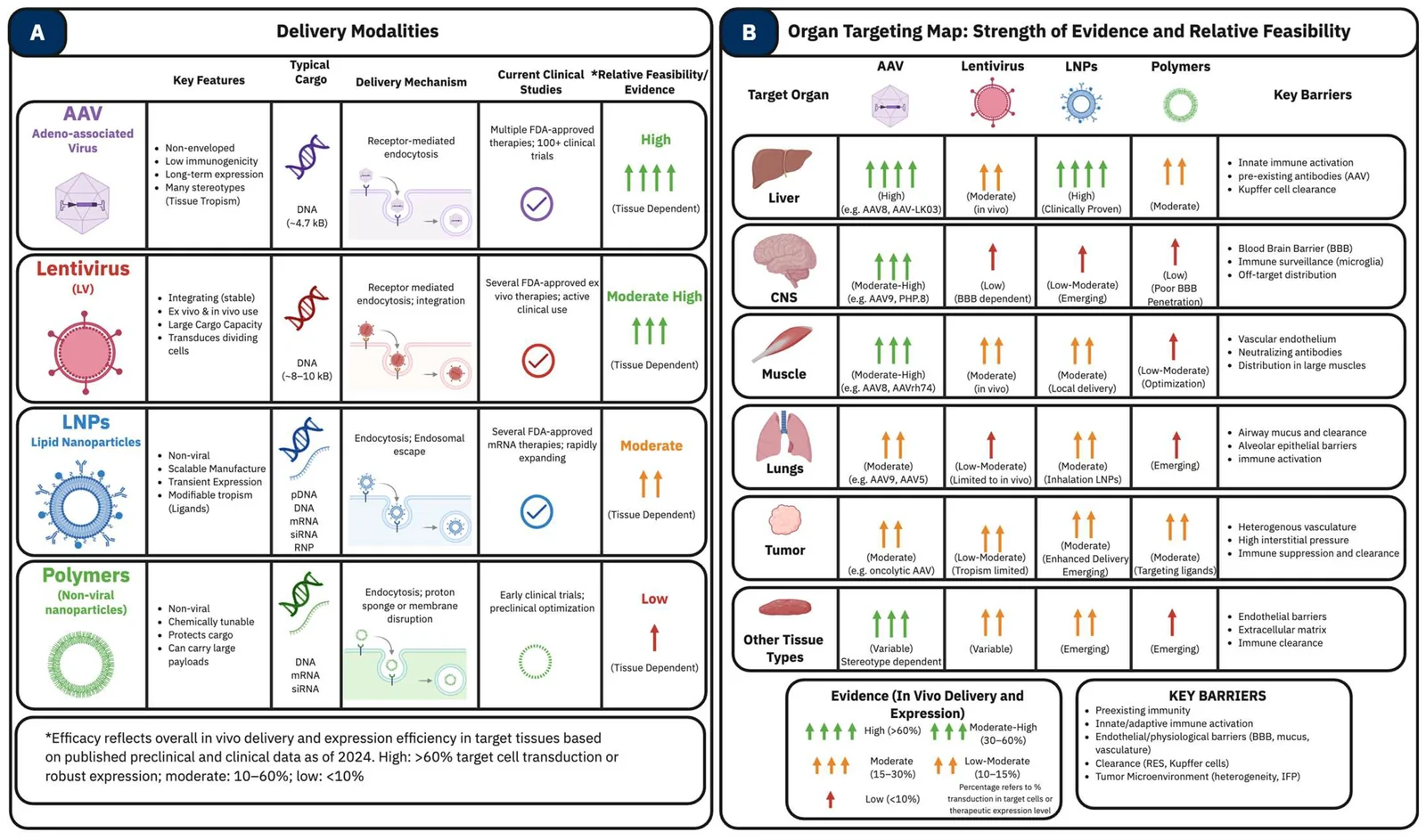
Comparative characteristics of major gene delivery modalities. Schematic comparison of viral and nonviral delivery platforms used in genetic medicine, including adeno-associated virus (AAV), lentiviral vectors, lipid nanoparticles (LNPs), and polymer-based systems. The figure summarizes relative differences in properties relevant to therapeutic development, including cargo capacity, durability of expression or activity, immunogenicity, manufacturability, repeat-dosing potential, and tissue or organ accessibility. AAV and lentiviral vectors provide clinically established approaches for durable gene transfer, whereas LNPs and other nonviral systems offer transient delivery that can be advantageous for RNA therapeutics and genome-editing components. Relative organ-specific performance is shown as a qualitative representation of current translational feasibility and the strength of available preclinical and clinical evidence rather than as a quantitative comparison of delivery efficiency across platforms. Delivery characteristics may vary substantially according to vector or particle composition, route of administration, cargo, dose, species, target cell type, and disease context. Accordingly, the figure is intended to illustrate broad platform-level trends and major delivery constraints rather than absolute or universally applicable rankings of efficacy. Abbreviations: AAV, adeno-associated virus; CNS, central nervous system; LNP, lipid nanoparticle.

Delivery efficiency remains a key determinant of whether a gene therapy platform can transition to clinical application [30,31,32,33,34,35,36,37,38,39,72,73,74,75,76,77,78,79,80,81]. The therapeutic utility of genome engineering technologies depends not only on the precision of the editing system itself but also on the ability to deliver nucleic acids, proteins, or ribonucleoprotein complexes into the appropriate cell population at therapeutically relevant levels [72,73,74,75,76,77,78,79,80,81]. Thus, high molecular editing efficiency cannot compensate for inadequate tissue or cell-type delivery. Consequently, delivery systems have evolved into active components of therapeutic design that influence tissue tropism, expression kinetics, immunogenicity, manufacturability, and clinical feasibility [30,31,32,33,34,35,36,37,38,39,74]. The principal delivery modalities and their relative characteristics are compared (Figure 3).

Adeno-associated virus (AAV) remains one of the most widely utilized delivery platforms in gene therapy because of its extensive clinical experience, ability to transduce non-dividing cells, and capacity to support long-term transgene expression [30,33,38,39]. Recombinant AAV vectors have enabled multiple approved therapies for retinal, neuromuscular, and systemic genetic disorders and remain widely used platforms for in vivo gene delivery [30,38,39]. Despite these successes, AAV-mediated delivery faces several important limitations [30,44,45,46,47,48]. The relatively small packaging capacity of AAV restricts delivery of large therapeutic payloads, including many base editors, prime editors, and complex regulatory circuits [30,64,70]. Furthermore, pre-existing neutralizing antibodies and adaptive immune responses against viral capsids can reduce efficacy, limit patient eligibility, and complicate repeat dosing strategies [44,45,46,47,48]. High systemic vector doses have also been associated with inflammatory responses and organ-specific toxicities, reinforcing the importance of dose optimization and immune monitoring during clinical development [45,46,47,48].

Engineering of AAV capsids is an active area of development aimed at improving therapeutic performance [30,31,32,33,34,35,36,37]. Strategies including directed evolution, rational design, peptide insertion, and high-throughput screening have been employed to enhance tissue tropism, increase transduction efficiency, and reduce off-target biodistribution [30,31,32,33,34,35,36,37]. These approaches are particularly important for tissues that are poorly transduced by naturally occurring AAV serotypes [30,37]. For example, engineered capsids with enhanced tropism for skeletal muscle, retinal tissue, and the central nervous system have demonstrated improved delivery efficiency while potentially reducing the vector doses required for therapeutic benefit [30,33,37]. Parallel efforts are focused on immune evasion through capsid engineering, transient immunosuppression, and strategies that reduce antigen presentation following vector administration [44,46,48].

Despite substantial progress, translation of engineered AAV performance from animal models to human patients remains challenging [31,32,34]. Species-specific differences in receptor expression, biodistribution, and immune recognition can significantly alter vector behavior and limit the predictive value of preclinical studies [31,34,36]. Consequently, improving translational predictability remains an important goal for next-generation AAV development [31,32].

Lentiviral vectors occupy a distinct niche within gene therapy because of their ability to integrate stably into the host genome and support long-term transgene expression [11,12,13,14]. This property has made lentiviral delivery particularly successful in ex vivo applications, including hematopoietic stem-cell modification and engineered immune-cell therapies [11,12,13,14,89,90,91,92,93]. In these settings, cells can be genetically modified under controlled manufacturing conditions and extensively characterized before reinfusion into patients [11,12,13,14,89,90,91,92,93]. Such control has contributed to the clinical use of lentiviral-based therapies for hemoglobinopathies, immunodeficiencies, and cellular immunotherapies [11,12,13,14,89,90,91,92,93]. Nevertheless, integration-associated risks such as insertional mutagenesis and clonal expansion remain important considerations requiring long-term monitoring [40,41,42].

Nonviral delivery systems are increasingly being developed as alternatives to viral vectors, particularly for RNA therapeutics and transient genome editing applications [72,73,74,75,76,77,78,79,80,81]. Among these technologies, lipid nanoparticles (LNPs) are among the most clinically mature nonviral platforms for nucleic acid delivery [72,73,74,75,76,77,78,79,80]. LNPs protect nucleic acid cargo from degradation, facilitate cellular uptake, promote endosomal escape, and can be modified to influence tissue targeting [72,73,74,75,76,77,78,79,80]. Their success in mRNA vaccines and RNA interference therapeutics has accelerated efforts to adapt them for delivery of CRISPR components, base editors, and prime editors [72,73,74,75,76,77,78,79,80]. A potential advantage of LNP-mediated delivery is transient cargo exposure and may lower the risk of off-target editing events [74,75,76,77].

The liver is currently the best-established target for systemic LNP-mediated nucleic-acid delivery [5,6,7,8,9,74,79]. Intravenously administered LNPs naturally accumulate in hepatocytes through interactions with serum proteins such as apolipoprotein E and uptake through hepatic receptors [74,79]. This intrinsic hepatotropism has enabled successful clinical translation of multiple RNA-based therapeutics and genome-editing strategies targeting liver disease [1,5,6,7,8,9,74]. However, this same property creates a major challenge for delivery to extrahepatic tissues [74,80,81].

To overcome these limitations, several approaches have been developed to redirect nanoparticle biodistribution [74,75,80,81]. Selective organ targeting (SORT) nanoparticles modify lipid composition to alter tissue accumulation profiles and improve delivery beyond the liver [74]. Additional strategies include surface ligand conjugation, peptide-based targeting systems, antibody-mediated targeting, and charge-altering lipid formulations [75,78,81]. These approaches have demonstrated encouraging results in targeting tissues such as the lung, spleen, endothelial cells, and immune-cell populations [74,75,80,81,82].

Polymer-based delivery systems represent another important class of nonviral technologies [72,74]. These materials can be engineered to condense nucleic acids, respond to specific biological environments, facilitate endosomal escape, and provide controlled cargo release [72,74]. Their chemical versatility allows extensive optimization of charge density, biodegradability, particle size, and targeting properties [72,74]. Although polymeric systems have generally lagged behind LNPs in clinical maturity, continued advances in polymer chemistry and hybrid nanoparticle design may improve their translational potential [72,74].

Organ-specific delivery remains one of the defining challenges of modern gene therapy [30,37,74]. The liver remains the most tractable target because of its vascular accessibility, fenestrated endothelium, and favorable uptake characteristics [5,6,7,8,9,74]. In contrast, delivery to the central nervous system is complicated by the blood–brain barrier and frequently requires specialized administration routes or engineered vectors capable of enhanced CNS penetration [32,33,37]. Skeletal muscle presents a different challenge because of its large tissue mass and requirement for widespread biodistribution, while pulmonary delivery must overcome mucus barriers, surfactant layers, and immune clearance mechanisms [30,80]. Solid tumors add further complexity through abnormal vasculature, elevated interstitial pressure, stromal barriers, and marked cellular heterogeneity [76].

These delivery challenges have important implications for therapeutic design [30,74]. For hepatic diseases, clinical feasibility has been demonstrated for several hepatic RNA and genome-editing applications [1,5,6,7,8,9,49,50,51]. In contrast, diseases affecting the central nervous system, skeletal muscle, lung, or solid tumors often remain limited by delivery constraints that influence platform selection and therapeutic performance [30,33,37,80]. Increasingly, successful gene therapy development depends on co-optimization of both the editing platform and the delivery technology rather than treating them as independent components [30,49,74].

Overall, delivery systems are evolving from broadly tropic carriers into highly engineered platforms capable of precise tissue and cell-type targeting [30,31,32,33,34,35,36,37,74]. AAV and lentiviral vectors continue to provide clinically used approaches for durable gene transfer, particularly in monogenic diseases and ex vivo cell engineering applications [11,12,13,14,38,39,89,90,91,92,93]. Meanwhile, LNPs and emerging nonviral technologies offer scalable, transient, and increasingly programmable alternatives that are especially well suited for RNA therapeutics and genome editing applications [5,6,7,8,9,72,73,74,75,76,77,78,79,80,81]. Broader application of gene therapy will require continued advances in extrahepatic delivery, repeat dosing, immune modulation, and matching of delivery kinetics to therapeutic mechanism [44,45,46,47,48,74,81].

## 2. A Multidimensional Design Framework for Modern Genetic Medicine

As gene therapy technologies have diversified, the field is increasingly defined not only by the ability to modify genetic material but by the capacity to control where, when, and to what extent a therapeutic effect occurs [10,15,16,17,18,19,20,21,22,23,24,25,26,27,28,29,74]. This multidimensional control is important for translating genome engineering and related technologies into clinically applicable therapies [10,15,16,17,18,19,20,21,22,23,24,25,26,27,28,29]. A useful framework for comparing platforms is therefore to consider three intersecting properties: genetic precision, temporal control, and dosage tunability [10,15,16,17,18,19,20,21,22,23,24,25,26,27,28,29]. These categories are intentionally ordinal and comparative rather than quantitative, and assignments reflect the predominant characteristics of current implementations rather than every possible platform configuration.

**Figure 4:**
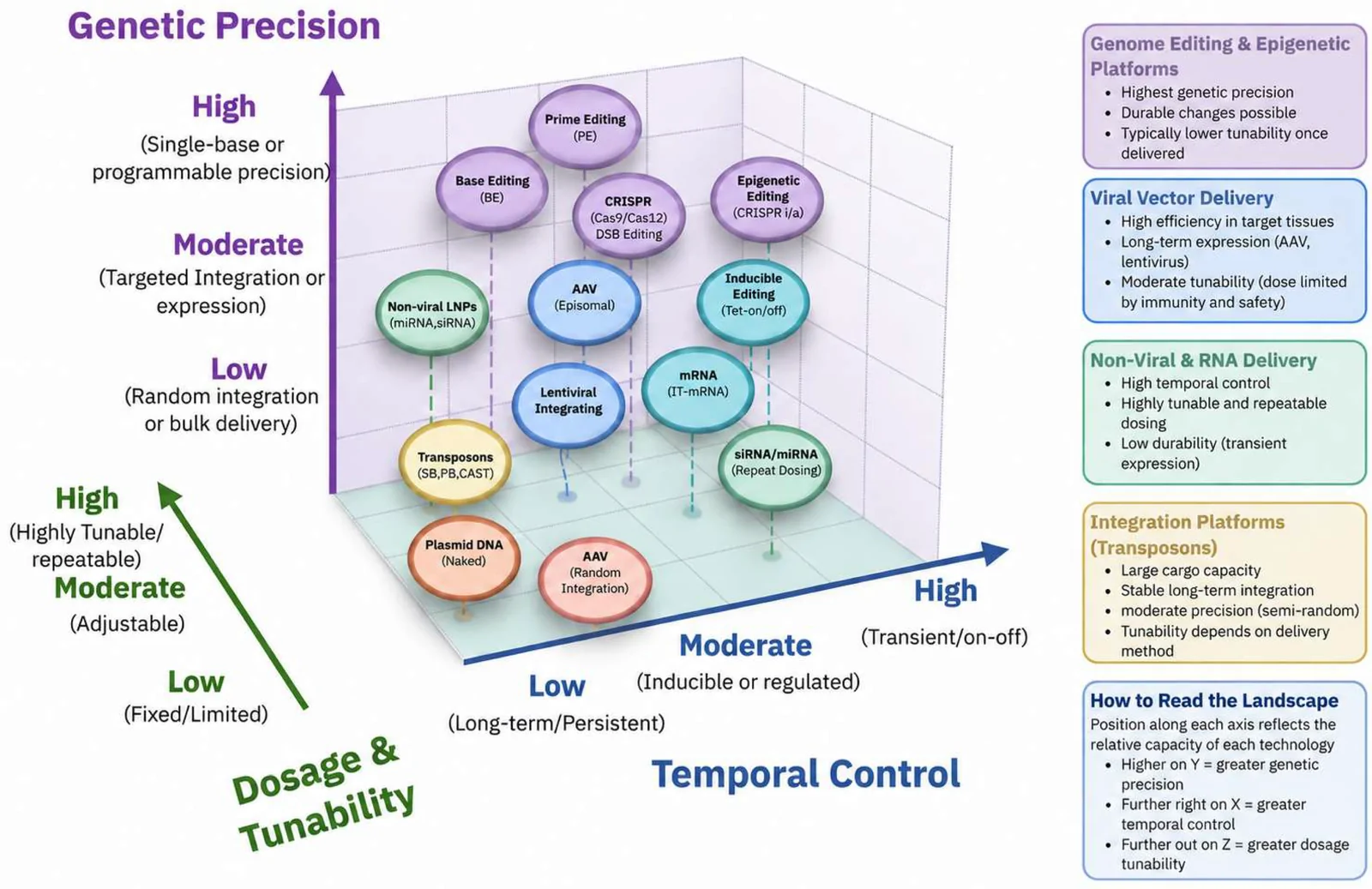
A Multidimensional Design Framework for Modern Gene Therapy. Genetic medicine platforms are positioned according to three intrinsic therapeutic properties: genetic precision, temporal control, and dosage tunability. Genetic precision reflects the ability to achieve the intended molecular and biological effect while limiting unintended genomic alterations; temporal control reflects the duration and reversibility of therapeutic activity; and dosage tunability reflects the extent to which therapeutic exposure or gene expression can be adjusted. Platforms shown include viral and nonviral gene delivery, CRISPR-based nuclease editing, base editing, prime editing, epigenetic editing, RNA therapeutics, and transposon-based integration systems. Relative placement is intended as a qualitative, conceptual comparison based on established mechanistic characteristics and available preclinical and clinical evidence rather than a quantitative ranking of therapeutic efficacy or safety. Table 1 provides a rubric for defining the scoring logic. Platform performance may vary according to editor design, cargo format, target sequence, cell type, delivery modality, and disease context. Abbreviations: AAV, adeno-associated virus; CRISPR, clustered regularly interspaced short palindromic repeats; CRISPRa, CRISPR activation; CRISPRi, CRISPR interference; LNP, lipid nanoparticle; mRNA, messenger RNA; siRNA, small interfering RNA; miRNA, microRNA; SB, sleeping beauty; PB, PiggyBac; CAST, CRISPR-associated transposase.

These axes represent intrinsic characteristics of the therapeutic intervention rather than absolute measures of superiority (Figure 4). Their clinical importance varies according to disease biology. A highly durable intervention may be advantageous for a severe monogenic disorder in which permanent correction is desirable, whereas greater reversibility and dose control may be preferred for common diseases, conditions with variable natural history, or targets with narrow therapeutic windows. Delivery efficiency and tissue specificity further constrain all three axes and should therefore be viewed as contextual determinants of whether the theoretical characteristics of a platform can be realized clinically. The scoring logic used to position these platforms is defined in the accompanying rubric (Table 1).

### 2.1. Genetic Precision

Genetic precision refers to the ability to produce the intended molecular and biological outcome while minimizing any unintended alterations elsewhere in the genome or at the intended target [18,19,20,21,22]. As genome editing has transformed from experimental systems to clinical application, characterization of unintended editing has become essential [18,19,20,21,22,40,41,42,43]. Off-target alterations may disrupt gene function or regulatory elements, whereas unintended on-target outcomes can include insertions or deletions, large deletions, chromosomal rearrangements, or other structural abnormalities [40,41,42,43]. This is particularly true of systems that utilize DSB-dependent editing [40,41,42,43].

Many strategies have been developed to improve targeting specificity [15,16,17,18,19,20,21,22]. Engineered high-fidelity Cas variants have been designed to reduce non-specific DNA interactions and thereby decrease off-target cleavage while retaining useful on-target activity [15,16,17]. Modifications to gRNA design, including truncated or chemically modified guides, can further alter sequence-recognition requirements [22]. Parallel advancements in computational prediction and genome-wide off-target detection methods, such as GUIDE-seq, CIRCLE-seq, and DISCOVER-seq, have improved the ability to identify potential off-target activity before clinical deployment [18,19,20,21,22].

Compared with conventional CRISPR endonucleases, base and prime editors can reduce reliance on canonical double-strand-break repair pathways [49,50,51,52,53,54,55,56,57,58,59,60,61,62,63,64,65,66,67,68,69,70,71]. However, these systems introduce their own precision considerations. Base editing can produce bystander nucleotide conversions or unwanted deamination, whereas prime editing can generate unintended products related to pegRNA activity, nicking, or cellular DNA-repair pathways [49,50,51,52,53,54,55,56,57,58,59]. Consequently, genetic precision should not be defined simply as the absence of off-target cleavage. Instead, it encompasses sequence specificity, the distribution of on-target products, structural genomic integrity, allelic selectivity where applicable, and the biological consequences of the resulting modification.

Genetic precision extends beyond molecular targeting to include biological context [10,15,16,17,18,19,20,21,22]. Editing a gene at its endogenous locus may preserve regulatory architecture and physiological expression patterns, while ectopic gene addition could bypass endogenous control mechanisms [10,11,12,13,14,15,16,17,18,19,20,21,22]. Conversely, highly precise sequence modification may provide limited therapeutic value if it occurs in the wrong cell population or at insufficient frequency. Precision is therefore increasingly understood as the combined specificity of the molecular intervention and its resulting physiological effect.

### 2.2. Temporal Control

Temporal control refers to the timing and duration of therapeutic activity [23,24,25,26,27,28,29,74,75,76,77]. This dimension is important because prolonged or uncontrolled activity of different genome engineering machinery can increase opportunities for unintended editing, immune recognition, or toxicity, while insufficient duration may limit therapeutic efficacy [40,41,42,43,44,45,46,47,48]. One strategy for controlling temporal exposure is through transient delivery systems [74,75,76,77]. For example, delivery of CRISPR components as mRNA or RNP complexes results in short-lived nuclease activity, which limits the window during which additional editing events can occur [74,75,76,77]. Similarly, RNA-based therapeutics including siRNAs and mRNA inherently provide transient effects due to rapid degradation and turnover within cells [5,6,7,8,9]. Their clinical effects can therefore often be adjusted through repeated administration.

In contrast, viral vectors such as AAV and lentiviruses can drive sustained or even permanent expression [11,12,13,14,30,31,32,33,34,35,36,37,38,39]. This can also be true of some transposases; Sleeping Beauty, for instance, has multiple integration effects, elevating the risk of off-target genomic modification events [83,84,85,86,87,88]. An important distinction must be made between the duration of the therapeutic machinery and the durability of the resulting genetic change. For example, transient exposure to a transposase or genome editor may nevertheless result in permanent genomic integration or sequence modification. Similarly, transient delivery of CRISPR or base-editing machinery can generate a durable biological effect because the resulting DNA modification persists after the editing components have been eliminated.

Long-term expression can be advantageous in diseases requiring continuous protein production, but can reduce flexibility after treatment [30,31,32,33,34,35,36,37,38,39,83,84,85,86,87,88]. Inducible expression systems, tissue-specific promoters, conditionally activated editors, and synthetic regulatory circuits have therefore been developed to introduce greater temporal control [23,28,29]. These approaches aim to align therapeutic activity with disease state, cellular context, or externally administered signals and may become increasingly important as gene therapy expands into conditions in which permanent constitutive activity is not desirable.

### 2.3. Dosage and Tunability

The third dimension, dosage and tunability, reflects the ability to control the magnitude of gene expression and therapeutic activity [5,6,7,8,9,23,28,29]. Therapeutic efficacy frequently depends on maintaining activity within an appropriate biological range [23,28]. Both insufficient and excessive activity can compromise the safety or effectiveness of the therapy [23,28].

Gene therapy platforms vary widely in their ability of dosage control [11,12,13,14,23,28]. In viral gene addition, vector dose, transduction efficiency, promoter activity, and vector copy number contribute to therapeutic expression [11,12,13,14]. For integrating vectors such as lentivirus, the vector copy number per cell can influence transgene expression and must be controlled in the context of insertion-related safety [40,41,42]. Similar considerations apply to transposon-based systems, in which transposase exposure may be transient but the resulting genomic integration is durable [83,84,85,86,87,88]. In non-integrating systems like AAV, episomal copy number and promoter strength govern the expression levels [30,31,32,33,34,35,36,37,38,39]. This system can be especially difficult to fine-tune once delivered [30,38,39].

A range of regulatory strategies have been developed to improve tunability [23,24,25,26,27,28,29]. These include the use of tissue-specific or inducible promoters, microRNA-regulated expression cassettes, feedback circuits, and regulatory elements capable of modulating transcriptional activity [23,24,25,26,27,28,29]. In genome editing, the dosage and duration of editing components influence both editing efficiency and the probability of unintended events [18,19,20,21,22,74,75,76,77].

RNA-based therapies offer a distinct advantage in this dimension because repeated dosing permits therapeutic exposure to be adjusted over time [5,6,7,8,9]. Epigenetic and transcriptional editing may also permit graded modulation of endogenous gene expression rather than permanent sequence alteration [23,24,25,26,27,28,29]. Although these platforms vary in their clinical maturity, they illustrate an important distinction between molecular precision and therapeutic tunability: the ability to make a highly specific permanent edit is not equivalent to the ability to adjust its effect after treatment.

### 2.4. Integration of the Three Axes

Taken together, genetic precision, temporal control, and dosage tunability define a multidimensional design space for modern genetic medicines [10,15,16,17,18,19,20,21,22,23,24,25,26,27,28,29]. No single platform maximizes all three axes simultaneously [5,6,7,8,9,10,15,16,17,18,19,20,21,22,23,24,25,26,27,28,29]. For example, DSB-based CRISPR editing offers high efficiency but requires careful characterization of both off-target and unintended on-target effects [10,15,16,17,18,19,20,21,22,40,41,42,43]. AAV-mediated gene addition provides durable expression in non-dividing cells but limited control over timing and dosage [30,31,32,33,34,35,36,37,38,39]. RNA therapeutics provide strong temporal control and repeat-dose tunability but generally require repeated treatment because their molecular effects are transient [5,6,7,8,9]. Transposon-based systems can produce durable genomic integration after transient delivery of the integration machinery, but insertion-site distribution and genomic safety remain important considerations [83,84,85,86,87,88]. Epigenetic editing may occupy an intermediate position by enabling potentially durable yet reversible or graded regulation without altering the underlying DNA sequence [23,24,25,26,27,28,29].

The location of a technology within this framework should be interpreted as context-dependent rather than absolute. Performance varies with editor design, cargo format, target sequence, cell type, delivery system, and disease biology. Figure 4 therefore represents a conceptual comparison based on established mechanistic characteristics and available preclinical and clinical evidence rather than a quantitative ranking of therapeutic superiority.

The continued evolution of gene therapy is increasingly focused on optimizing across these dimensions simultaneously [10,15,16,17,18,19,20,21,22,23,24,25,26,27,28,29,74]. Advances in high-fidelity editing enzymes, inducible and self-regulating expression systems, and targeted delivery technologies are being developed to improve control over the sequence, timing, location, and magnitude of therapeutic activity [15,16,17,18,19,20,21,22,23,24,25,26,27,28,29,74,75,76,77,78,79,80,81]. This multidimensional optimization may be particularly important as gene therapy expands into common diseases, where treatment of larger and potentially healthier populations requires increasingly stringent safety margins, predictable durability, and control of therapeutic exposure.

### 2.5. Clinical Maturity and Evidence Hierarchy

The expanding number of genetic technologies makes it increasingly important to distinguish technological capability from clinical maturity. Platforms that demonstrate high editing efficiency in cultured cells or animal models should not be interpreted as equivalent to technologies supported by human safety data, durable clinical outcomes, or regulatory approval. The current genetic medicine landscape spans a continuum from established approved modalities to first-in-human interventions and early preclinical technologies. The evidence for clinical maturity across genetic medicines is summarized in the accompanying hierarchy (Table 2).

Viral gene transfer and engineered cellular therapies have substantial clinical experience relative to newer genome-editing platforms [11,12,13,14,38,39,89,90,91,92,93]. Lentiviral hematopoietic stem-cell therapies, AAV-based in vivo gene therapies, and genetically engineered immune-cell products have established that durable genetic modification can produce clinically meaningful benefit across several disease classes [11,12,13,14,38,39,89,90,91,92,93]. CRISPR nuclease-based editing has now achieved regulatory approval in ex vivo hematopoietic stem-cell therapy [2,3].

Other platforms occupy earlier but rapidly advancing stages of translation. In vivo CRISPR editing targeting transthyretin established proof of principle for systemic nonviral delivery of genome-editing components to human hepatocytes 1. Base editing has now advanced from extensive preclinical development to clinical evidence in humans. In a phase 1 study, a single administration of VERVE-102, an LNP-delivered adenine base-editing therapy targeting hepatic PCSK9, produced dose-dependent reductions in circulating PCSK9 and LDL cholesterol in treated participants [94]. These effects remained evident during available follow-up, including at least one year in a subset of participants, although broader conclusions regarding long-term safety and clinical cardiovascular benefit will require continued study.

Prime editing has similarly entered early clinical translation. PM359 uses ex vivo prime editing of autologous CD34+ hematopoietic stem cells to correct a recurrent NCF1 deletion responsible for p47phox-deficient chronic granulomatous disease. Initial clinical reporting in two participants demonstrated hematopoietic engraftment and measurable restoration of NADPH oxidase activity [95]. These findings represent an important proof of principle but remain early clinical evidence and should not be interpreted as establishing long-term efficacy or safety.

Epigenetic editing, programmable transposases, CRISPR-associated integration systems, and several targeted extrahepatic delivery technologies remain earlier in development [23,24,25,26,27,28,29,74,75,76,77,78,79,80,81,82,83,84,85,86,87,88]. These platforms enable distinct forms of gene regulation or integration, but remain dependent on demonstration of reproducible delivery, safety, manufacturing consistency, and clinical benefit.

Clinical maturity therefore represents an additional interpretive layer over the three-axis framework described above. A platform may theoretically provide superior precision or tunability yet remain less clinically actionable than a more established technology with a well-characterized manufacturing process and safety profile. Comparison among genetic medicines should therefore consider both what a platform is capable of doing and the strength of evidence demonstrating that it can do so safely and reproducibly in humans.

## 3. Clinical Landscape: Current Applications and Expansions

**Figure 5:**
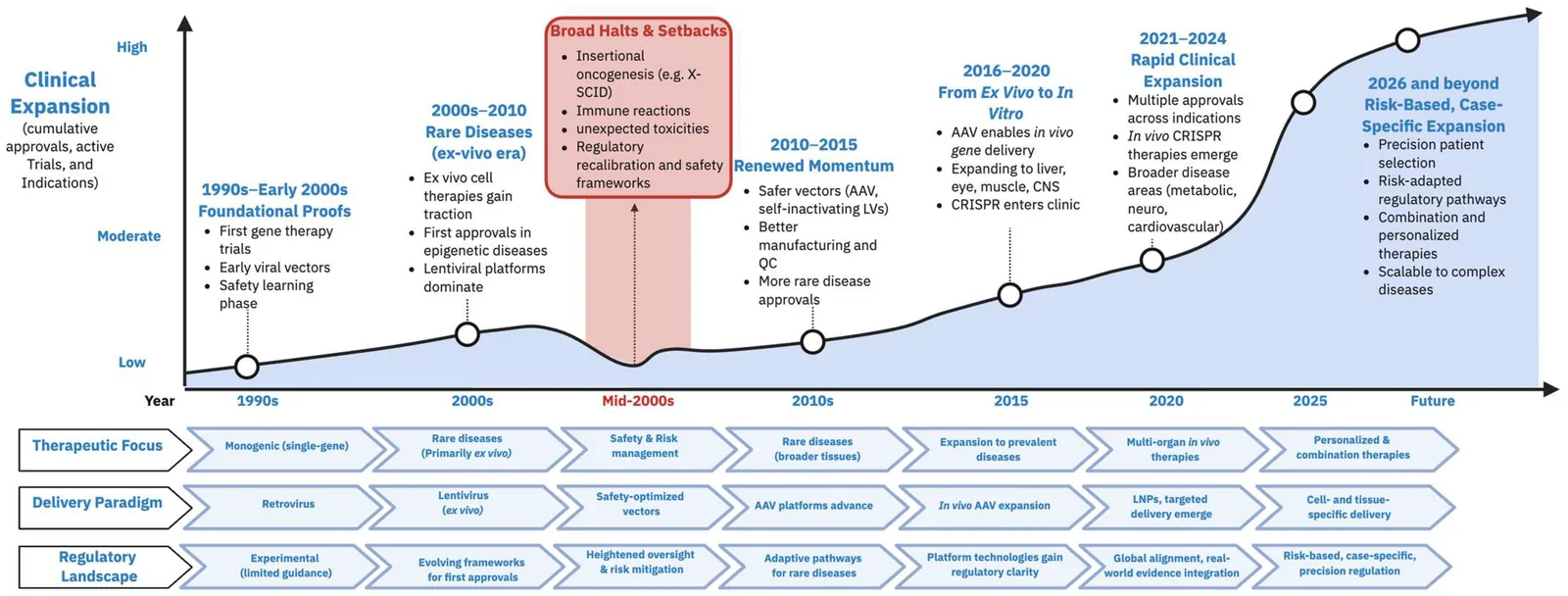
Clinical expansion of gene therapy and genetic medicine. Schematic representation of the evolving clinical landscape of genetic medicine, from initial applications in rare monogenic disorders to ex vivo genetically modified cell therapies, in vivo gene delivery and genome editing, and broader investigation in oncologic, cardiovascular, metabolic, and other disease settings. Early clinical development was concentrated in severe disorders with well-defined genetic etiologies and high unmet medical need, whereas advances in genome engineering, delivery technologies, manufacturing, and clinical experience have enabled evaluation of genetic interventions across increasingly diverse indications. The figure illustrates expansion in therapeutic scope rather than a strictly linear chronology or replacement of earlier approaches by newer technologies; multiple modalities continue to develop in parallel and differ substantially in clinical maturity, delivery requirements, durability, and benefit-to-risk considerations.

The clinical landscape of gene therapy has undergone substantial changes over the past decade [1,2,3,4,5,6,7,8,9]. The field now includes an increasing number of approved and clinical-stage products across expanding indications [1,2,3,4,11,12,13,14,89,90,91,92,93]. This regulatory trajectory has not been linear, marked by periods of rapid progress, safety-related halts, and slow-moving cautionary periods [11,12,13,14,40,41,42,43,44,45,46,47,48]. These together have shaped the rigorous and risk-aware development paradigm of gene therapies [40,41,42,43,44,45,46,47,48]. The expansion of genetic medicine across these clinical settings is summarized schematically (Figure 5).

As stated, the early clinical development of gene therapy was previously primarily focused on rare monogenic disease [11,12,13,14]. These disease contexts were defined by clear genetic etiology and well-defined therapeutic targets that provided a favorable risk-to-benefit profile [11,12,13,14]. Initial success in these monogenic diseases established a valid proof of concept that genetic diseases could be treated and, in some cases, produce durable clinical benefit and, in selected settings, potentially curative outcomes, through gene therapy [11,12,13,14]. These indications were especially well-suited for the early gene therapy approaches because the modest restoration of gene function would yield significant clinical benefit [11,12,13,14]. The field, however, also experienced notable clinical setbacks that have informed current development strategies [40,41,42,43,44,45,46,47,48]. The adverse effects during this time, such as immune responses, vector-associated toxicities, and insertional mutagenesis, led to temporary halts in clinical trials as well as heightened regulatory scrutiny [40,41,42,43,44,45,46,47,48]. The events of these trials have driven the adoption of more comprehensive preclinical evaluation, improved vector design, and enhanced patient monitoring [40,41,42,43,44,45,46,47,48]. As a result, contemporary gene therapy development is guided by regulation that is both risk-based and case-specific [40,41,42,43,44,45,46,47,48]. Regulation of therapeutics is tailored to disease severity, benefit-to-risk analysis, target tissue, and acceptable safety thresholds [40,41,42,43,44,45,46,47,48].

An important development has been the expansion from ex vivo modification to in vivo therapeutic approaches [1,2,3,4,11,12,13,14,89,90,91,92,93]. Ex vivo approaches, in which cells are isolated, genetically modified, and reinfused into the patient, were among the first to achieve clinical success and now define the field of cell therapy [11,12,13,14,89,90,91,92,93]. This principle is exemplified by lentiviral-modified hematopoietic stem cells for hemoglobinopathies and chimeric antigen receptor (CAR) T cell therapies for hematologic malignancies [11,12,13,14,89,90,91,92,93]. These strategies benefit from very controlled and quality-assessed gene modification as well as a lower risk of systemic exposure to gene delivery vectors [11,12,13,14,89,90,91,92,93]. The ex vivo modification, however, is associated with high costs, complex manufacturing, and logistical challenges [11,12,13,14,89,90,91,92,93].

Contrasting this, in vivo gene therapy aims to deliver therapeutic cargo directly to tissues within the body [1,5,6,7,8,9,49,50,51,72,73,74,75,76,77,78,79,80,81]. This approach can reduce some of the cell-processing and logistical requirements associated with ex vivo approaches, although scalability remains dependent on delivery, manufacturing, and indication [1,5,6,7,8,9,72,73,74,75,76,77,78,79,80,81]. In vivo gene editing is particularly attractive in diseases affecting organs such as the liver, muscle, or CNS [1,5,6,7,8,9,49,50,51,72,73,74,75,76,77,78,79,80,81]. Recent advances in delivery technologies and genome editing have enabled in vivo therapies to achieve clinical-grade outcomes, particularly in hepatocyte-targeted applications, with therapies being developed for oncological purposes as well [1,5,6,7,8,9,49,50,51,72,73,74,75,76,77,78,79,80,81]. Interest in in vivo approaches partly reflects the potential to avoid ex vivo cell collection, manipulation, and reinfusion [1,5,6,7,8,9,72,73,74,75,76,77,78,79,80,81]. However, challenges related to delivery specificity, immune responses, and dose control remain a primary concern for clinical application [30,44,45,46,47,48,74,75,76,77,78,79,80,81].

Approved and late-stage clinical gene therapies continue to concentrate within a few key areas touched on previously [1,2,3,4,11,12,13,14,96]. Genetically engineered cellular therapies have also become established components of treatment for selected hematologic malignancies [89,90,91,92,93]. Engineered cell therapies, particularly CAR-T cells, have demonstrated the ability to reprogram immune cells to recognize and eliminate cancer cells, achieving durable remissions in certain hematologic malignancies [89,90,91,92,93]. These oncological approaches represent a convergence of gene therapy and immunotherapy, and ongoing efforts are focused on improving efficacy in solid tumors, enhancing persistence, and reducing toxicity [89,90,91,92,93].

Gene therapy has also begun to move beyond rare monogenic diseases into complex and more prevalent disorders such as cardiovascular and metabolic disease [5,6,7,8,9,49,50,51,52,53]. This expansion represents an important change in therapeutic context. For rare, severe genetic disorders with limited treatment alternatives, substantial procedural complexity or uncertain long-term risk may be acceptable when balanced against the natural history of the disease. In contrast, interventions intended for common chronic diseases must meet substantially different expectations for scalability, manufacturing, cost, and long-term safety.

In vivo editing of PCSK9 provides an important example of this transition. Preclinical studies demonstrated that base editing of hepatic PCSK9 could durably reduce circulating LDL cholesterol in nonhuman primates [49,50,51]. This strategy has now advanced into human clinical testing. VERVE-102, an investigational adenine base-editing therapy delivered through a hepatocyte-targeted lipid nanoparticle, was evaluated in a phase 1 single-ascending-dose study involving adults with heterozygous familial hypercholesterolemia or premature coronary artery disease [94]. Treatment provides clinical evidence that in vivo base editing can produce dose-dependent reductions in PCSK9 and LDL cholesterol over the available follow-up period.

The significance of this development extends beyond PCSK9 itself. Unlike many early gene therapy indications, hypercholesterolemia affects a large patient population for whom multiple effective conventional therapies are already available. The acceptable risk threshold is therefore fundamentally different from that of a severe, otherwise untreatable monogenic disorder. Expansion of permanent genome editing into common disease will require particularly strong evidence of genomic safety, predictable biodistribution, durable therapeutic effect, and favorable long-term benefit-to-risk balance.

At the same time, clinical translation of prime editing demonstrates the movement of precise sequence rewriting from preclinical models into human therapy. PM359 applies prime editing ex vivo to autologous hematopoietic stem cells in p47phox-deficient chronic granulomatous disease and has produced early evidence of restored cellular function following transplantation [95]. Together, these developments illustrate that the clinical landscape is no longer defined simply by whether genome editing can be performed in humans, but increasingly by which editing mechanism, delivery strategy, and degree of permanence are most appropriate for a particular disease.

These emerging indications introduce new challenges, including the need for highly reproducible manufacturing, comprehensive genomic safety assessment, scalable delivery, and long-term monitoring [40,41,42,43,44,45,46,47,48,74,75,76,77,78,79,80,81]. As genetic medicines move from rare diseases toward larger populations, therapeutic development will increasingly depend on matching the degree of intervention to disease severity and on demonstrating that the expected durability of benefit justifies the irreversibility or persistence of the genetic modification.

### 3.1. Regulatory Evolution

Regulation of gene therapy has evolved in parallel with technological advances and accumulated clinical experience [1,2,3,4,10,15,16,17,18,19,20,21,22,23,24,25,26,27,28,29,40,41,42,43,44,45,46,47,48,49,50,51,52,53,54,55,56,57,58,59,60,61,62,63,64,65,66,67,68,69,70,71]. Early regulatory oversight was shaped by substantial uncertainty regarding immune activation, vector-associated toxicity, insertional mutagenesis, and the long-term consequences of permanent genetic modification [40,41,42,43,44,45,46,47,48]. These experiences established a cautious development paradigm emphasizing extensive preclinical evaluation, conservative dose escalation, and long-term patient monitoring.

As the field has matured, regulation has increasingly become risk-based, mechanism-informed, and product-specific rather than uniform across all gene therapy platforms. Therapeutic products are evaluated in the context of their molecular mechanism, delivery modality, target tissue, persistence, manufacturing characteristics, disease severity, and expected benefit-to-risk profile. This approach recognizes that an ex vivo edited hematopoietic stem-cell product, an intravenously delivered AAV, and a transient LNP-delivered genome editor present substantially different biological and clinical risks.

For genome-editing products in particular, regulatory evaluation increasingly emphasizes characterization of both intended and unintended genomic outcomes. The FDA’s 2024 guidance for human gene therapy products incorporating genome editing addresses product design, manufacturing and testing, nonclinical safety evaluation, and clinical trial design [97]. It emphasizes assessment of off-target editing as well as unintended changes occurring at the intended genomic locus. This distinction is important because genomic safety cannot be established solely by demonstrating sequence-specific targeting; structural variants, large deletions, rearrangements, product heterogeneity, and other unintended outcomes can also arise at the intended editing site [40,41,42,43].

Regulatory expectations are continuing to evolve as analytical technologies improve. In 2026, the FDA issued draft guidance specifically addressing the use of next-generation sequencing methods in nonclinical safety assessment of human genome-editing products [98]. The guidance provides recommendations related to sequencing strategies, sample selection, bioinformatic analysis, off-target evaluation, and assessment of genomic integrity, reflecting the increasing importance of standardized and sensitive methods for characterizing unintended editing outcomes. Such developments are particularly relevant as base editing, prime editing, multiplex editing, and other technologies create increasingly complex classes of potential genomic products.

At the same time, regulatory science is beginning to consider how knowledge can be transferred across related genome-editing programs. A 2026 FDA draft guidance describes circumstances in which public or platform knowledge may potentially be leveraged across chemistry, manufacturing and controls, nonclinical, and clinical development programs [99]. This approach may become particularly important for rare and individualized genetic diseases, where conventional development programs can be constrained by small patient populations and the need to develop multiple related products efficiently. Importantly, leveraging platform knowledge does not eliminate the requirement to establish the relevance of prior data to a new product, target, delivery system, or patient population.

Long-term follow-up remains a central component of gene therapy development because both vector-mediated and genome-editing interventions can produce biological consequences that persist long after administration 73–81. The duration and intensity of follow-up should therefore reflect the mechanism of action, permanence of the genetic alteration, cell population affected, potential for clonal expansion, and availability of methods for identifying delayed adverse outcomes.

Although specific regulatory pathways, submission requirements, and oversight structures vary across jurisdictions, major agencies including the U.S. Food and Drug Administration (FDA), European Medicines Agency (EMA), Medicines and Healthcare products Regulatory Agency (MHRA), and other national regulatory authorities share several core priorities in the evaluation of gene and genome-editing therapies. These include characterization of genomic integrity and unintended genetic alterations, assessment of biodistribution and immunogenicity, demonstration of manufacturing consistency and product quality, and appropriate long-term follow-up for delayed or persistent adverse effects. Across regulatory systems, the acceptable level of uncertainty is also evaluated in relation to disease severity, available treatment alternatives, expected durability of benefit, and the permanence or reversibility of the intervention. Thus, while specific development pathways and evidentiary requirements differ among regulatory authorities, contemporary oversight of genetic medicines is increasingly aligned around a risk-based, mechanism-informed assessment of product quality, genomic safety, durability, and overall benefit-to-risk balance.

Overall, regulatory evolution in gene therapy reflects a transition from broad caution toward increasingly mechanistic and evidence-based assessment. Rather than indicating that safety requirements have become less stringent, this transition reflects an improved ability to identify the distinct risks associated with different technologies. Continued alignment among genome engineering, analytical genomics, manufacturing science, long-term clinical follow-up, and regulatory standards will be essential as genetic medicines expand into larger and more diverse patient populations.

### 3.2. Matching Platform to Disease Context

The expanding range of genetic medicine platforms makes therapeutic selection increasingly disease-specific. No single technology is inherently optimal across all indications. Instead, the appropriate platform depends on the genetic mechanism of disease, target-cell biology, required durability, available delivery route, therapeutic window, and consequences of unintended modification.

For loss-of-function monogenic diseases, stable gene addition may remain appropriate when precise regulation of the endogenous locus is not required and sufficient expression can be achieved safely [11,12,13,14,30,31,32,33,34,35,36,37,38]. Direct sequence correction may offer an advantage when restoration of endogenous regulation is biologically important or when a pathogenic variant can be efficiently modified [10,15,16,17,18,19,20,21,22,49,50,51,52,53,54,55,56,57,58,59,60,61,62,63,64,65,66,67,68,69,70,71]. Gene disruption can be particularly effective when therapeutic benefit results from reducing a disease-associated protein or regulatory pathway, as demonstrated by BCL11A-targeted approaches in hemoglobinopathies and TTR- or PCSK9-targeted approaches in the liver [1,2,3,49,50,51,94].

The desired duration of therapy is equally important. Severe lifelong disorders may justify permanent genomic modification when the expected benefit substantially outweighs uncertainty associated with irreversibility. In diseases in which therapeutic requirements change over time, or in which the target pathway has a narrow safety margin, transient or adjustable approaches may be preferable. RNA therapeutics and inducible regulatory systems can provide greater dose and temporal control at the cost of repeated treatment or more complex regulation [5,6,7,8,9,23,24,25,26,27,28,29].

Target tissue frequently represents the practical limiting factor. The liver is currently among the most tractable organs for systemic nonviral nucleic-acid delivery [5,6,7,8,9,74,79,94], whereas the central nervous system, skeletal muscle, lung, and solid tumors present substantially different anatomical and biological barriers [30,32,33,37,80]. Consequently, the theoretical precision of an editing technology may be less clinically important than the ability to deliver it safely and uniformly to the disease-relevant cell population.

Finally, acceptable risk depends strongly on disease severity and available alternatives. A permanent intervention may have an acceptable benefit-to-risk profile in a severe disorder with high morbidity and limited therapeutic options but require a substantially higher level of safety evidence before application to a common condition that can already be managed with effective conventional therapies. Thus, platform selection should be regarded as an optimization problem rather than a technological hierarchy: the goal is not to identify the most advanced editing system, but to identify the combination of mechanism, delivery, durability, and controllability that best matches the clinical problem. As a practical example, sickle cell disease favors ex vivo editing because hematopoietic stem and progenitor cells can be isolated, modified, quality-tested, and reinfused; the resulting permanent edit is also compatible with the need for durable correction. By contrast, a chronic liver disorder with an established RNA target may favor a repeat-dosed RNA therapeutic when reversibility and dose adjustment are more important than permanent sequence correction. In this way, the framework is intended to narrow platform choice according to disease requirements rather than to rank technologies universally.

## 4. Conclusions

The field of gene therapy has undergone a substantial transformation over the past several decades, evolving from early viral-mediated gene addition to a diverse collection of technologies capable of adding, editing, regulating, and rewriting genetic information [10,11,12,13,14,15,16,17,18,19,20,21,22,23,24,25,26,27,28,29,49,50,51,52,53,54,55,56,57,58,59,60,61,62,63,64,65,66,67,68,69,70,71]. Advances in CRISPR-Cas systems, base editing, prime editing, epigenetic regulation, RNA therapeutics, and programmable integration have expanded the range of genetic interventions that can be investigated therapeutically [5,6,7,8,9,10,15,16,17,18,19,20,21,22,23,24,25,26,27,28,29,49,50,51,52,53,54,55,56,57,58,59,60,61,62,63,64,65,66,67,68,69,70,71,83,84,85,86,87,88]. Several of these approaches have now progressed from molecular proof of concept to clinical application, while others remain at earlier stages of translational development [1,2,3,4,89,90,91,92,93,94,95,96].

A central theme emerging from this evolution is that therapeutic performance cannot be defined by editing efficiency or molecular precision alone. Genetic precision, temporal control, and dosage tunability represent distinct properties that must be considered together with delivery efficiency, tissue accessibility, durability, manufacturing, and clinical maturity. No current platform simultaneously maximizes all of these characteristics. Instead, each technology creates a different balance among permanence, controllability, delivery feasibility, and risk.

This framework also changes how technological progress should be interpreted. The transition from gene addition to genome editing does not necessarily represent replacement of older technologies by newer ones. Viral gene transfer remains highly appropriate for selected diseases requiring durable expression; RNA therapeutics provide advantages when repeat dosing and reversibility are desirable; nuclease editing enables efficient disruption or modification of endogenous loci; base and prime editing expand the range of precise sequence changes; and epigenetic or transcriptional systems may provide additional control without permanent sequence alteration. The clinical value of each approach is therefore determined by its fit to the biological and therapeutic requirements of the disease.

Recent clinical advances illustrate the practical relevance of these platform-specific trade-offs. Approved CRISPR-edited cellular therapies have demonstrated that targeted genome modification can produce clinically meaningful benefit in selected indications [2,3]. In vivo CRISPR editing has demonstrated systemic editing of human hepatocytes [1], while recent clinical studies of PCSK9 base editing and ex vivo prime editing show that additional editing architectures are now entering human translation [94,95]. These developments support evaluation of genome-editing approaches in additional disease settings beyond genetic intervention but also increase the importance of platform-specific safety evaluation and long-term follow-up.

Significant challenges remain. Efficient delivery to nonhepatic tissues, mitigation of immune responses, characterization of unintended genomic outcomes, control of long-term biological effects, and scalable manufacturing continue to limit broader clinical implementation [30,31,32,33,34,35,36,37,38,39,40,41,42,43,44,45,46,47,48,74,75,76,77,78,79,80,81]. Regulatory evolution increasingly reflects these challenges through mechanism-specific evaluation of genome integrity, manufacturing, biodistribution, and durability [97,98,99].

The next phase of genetic medicine will therefore be defined not simply by the development of editors capable of making increasingly complex genetic changes, but by the ability to select and control those technologies according to clinical need. Continued integration of genome engineering, delivery science, computational design, immunology, manufacturing, and regulatory science will determine how broadly these therapies can be applied. Within this context, a central challenge is determining which genetic modification, delivered to which cells, for what duration and at what magnitude, provides the most favorable therapeutic outcome for a particular disease.

## Figures and Tables

**Table 1:** Rubric to define scoring logic for Figure 4.

Dimension	Low	Moderate	High
Genetic precision	Random/broad genomic or cellular effect	Targeted locus/expression	Defined nucleotide or programmable locus with characterized product profile
Temporal control	Persistent/non-reversible	Regulated/inducible	Transient or readily discontinued
Tunability	Fixed after administration	Adjustable before administration	Repeat-dose or dynamically adjustable
Delivery maturity	Limited/preclinical	Tissue-restricted clinical feasibility	Validated clinical delivery
Clinical maturity	Preclinical	Clinical-stage	Approved/established

**Table 2:** Clinical maturity and evidence hierarchy for genetic medicines.

Platform	Therapeutic Mechanism	Permanence	Temporal Control	Tunability	Key Safety Concern	Delivery Constraint	Clinical Maturity
AAV gene addition	transgene expression	durable	low	low	immunity/toxicity	tissue tropism	approved
Lentiviral	genomic integration	permanent	low	low	insertional risk	predominantly ex vivo	approved
CRISPR nuclease	disruption/correction	permanent	machinery transient	low after editing	DSB/SV risk	tissue dependent	approved/clinical
Base editing	nucleotide conversion	permanent	machinery transient	low after editing	bystander/off-target edits	currently strongest in liver/ex vivo	clinical
Prime editing	sequence rewriting	permanent	machinery transient	low after editing	product heterogeneity/repair effects	major delivery burden	early clinical
RNA therapeutics	RNA/protein modulation	transient	high	high	repeated exposure	tissue dependent	approved
Epigenetic editing	expression regulation	variable	moderate-high	potentially high	persistence/off-target regulation	early delivery	preclinical/early
CAST/transposons	genomic integration	permanent	enzyme transient	low after integration	insertion profile	delivery/cargo	mainly preclinical

## Data Availability

No new data were created or analyzed in this study. Data sharing is not applicable to this article.

## Abbreviations

AAV	Adeno-associated virus
CAR	Chimeric antigen receptor
CAR-T	Chimeric antigen receptor T-cell
Cas	CRISPR-associated protein
CAST	CRISPR-associated transposase
CNS	Central nervous system
CRISPR	Clustered regularly interspaced short palindromic repeats
CRISPRa	CRISPR activation
CRISPRi	CRISPR interference
DNA	Deoxyribonucleic acid
DSB	Double-strand break
EMA	European Medicines Agency
FDA	U.S. Food and Drug Administration
gRNA	Guide RNA
HDR	Homology-directed repair
HR	Homologous recombination
LNP	Lipid nanoparticle
LDL	Low-density lipoprotein
mRNA	Messenger RNA
MHRA	Medicines and Healthcare products Regulatory Agency
NADPH	Nicotinamide adenine dinucleotide phosphate
NHEJ	Non-homologous end joining
pegRNA	Prime-editing guide RNA
RNA	Ribonucleic acid
RNAi	RNA interference
RNP	Ribonucleoprotein
RT	Reverse transcriptase
shRNA	Short hairpin RNA
siRNA	Small interfering RNA
SORT	Selective organ targeting
TALEN	Transcription activator-like effector nuclease
TTR	Transthyretin
ZFN	Zinc finger nuclease
